# Pharmacy Naloxone Standing Order and Community Opioid Fatality Rates Over Time

**DOI:** 10.1001/jamanetworkopen.2024.27236

**Published:** 2024-08-29

**Authors:** Ziming Xuan, Alexander Y. Walley, Shapei Yan, Avik Chatterjee, Traci G. Green, Robin A. Pollini

**Affiliations:** 1 Department of Community Health Sciences, Boston University School of Public Health, Boston, Massachusetts; 2 Department of Epidemiology, Boston University School of Public Health, Boston, Massachusetts; 3 Grayken Center for Addiction, Clinical Addiction Research and Education Unit, Section of General Internal Medicine, Department of Medicine, Boston Medical Center and Boston University School of Medicine, Boston, Massachusetts; 4 Institute for Behavioral Health, The Heller School for Social Policy and Management at Brandeis University, Waltham, Massachusetts; 5 Department of Behavioral Medicine and Psychiatry, West Virginia University School of Medicine, Morgantown; 6 Department of Epidemiology and Biostatistics, West Virginia University School of Public Health, Morgantown

## Abstract

**Importance:**

Despite the proliferation of pharmacy standing-order naloxone dispensing across many US states before the change to over-the-counter status, few policy analyses have evaluated the implementation of pharmacy naloxone standing orders in addressing opioid overdose fatality among communities.

**Objective:**

To determine whether the implementation of pharmacy standing-order naloxone was associated with lower opioid fatality rates compared with communities without pharmacies with standing-order naloxone.

**Design, Setting, and Participants:**

This retrospective multisite study was conducted with an interrupted time series analysis across 351 municipalities in Massachusetts over 24 quarters (from January 1, 2013, through December 31, 2018). Standing-order naloxone dispensing data were collected from 2 sources for all major chain pharmacies and many independent pharmacies, covering 70% of retail pharmacies in Massachusetts. Municipalities had various standing-order naloxone implementation inceptions during the study period. Data were analyzed from December 2021 to November 2023.

**Exposure:**

The main exposure was measured by the first quarter with standing-order naloxone dispensation as the actual implementation inception.

**Main Outcomes and Measures:**

The primary study outcome was municipal opioid fatality rate per 100 000 population obtained from the Massachusetts Registry of Vital Records and Statistics.

**Results:**

The median (IQR) population size across 351 municipalities was 10 314 (3635 to 21 781) people, with mean (SD) proportion of female individuals was 51.1% (2.8 percentage points). Pharmacies from 214 municipalities (60.9%) reported dispensing standing-order naloxone over the study period. At the baseline of the first quarter of 2013, municipalities that eventually had standing-order naloxone had greater quarterly opioid fatality rates compared with those that never implemented standing-order naloxone (3.51 vs 1.03 deaths per 100 000 population; *P* < .001). After adjusting for municipal-level sociodemographic and opioid prevention factors, there was significant slope decrease of opioid fatality rates (annualized rate ratio, 0.84; 95% CI, 0.78-0.91; *P* < .001) following standing-order naloxone dispensing, compared with the municipalities that did not implement standing-order naloxone. There were no significant level changes of opioid fatality rates in the adjusted models. Sensitivity analyses yielded similar and significant findings.

**Conclusions and Relevance:**

These findings suggest that community pharmacy dispensing of naloxone with standing orders was associated with a relative, gradual, and significant decrease in opioid fatality rates compared with communities that did not implement the standing-order naloxone program. These findings support the expansion of naloxone access, including over-the-counter naloxone as part of a multifaceted approach to address opioid overdose.

## Key Points

**Question:**

Was community implementation of pharmacy standing-order naloxone dispensing associated with municipal opioid fatality rates across municipalities in Massachusetts?

**Findings:**

In this study with interrupted time series analysis of all 351 Massachusetts municipalities, communities with pharmacies implementing standing-order naloxone dispensing was associated with reduced opioid fatality rates over time compared with municipalities that did not implement standing-order naloxone dispensing.

**Meaning:**

These findings of reduced opioid fatality rates associated with standing-order naloxone dispensing support the expansion of community naloxone access, including over-the-counter naloxone, as part of a multifaceted approach to address opioid overdose.

## Teaser

This cross-sectional study examines whether implementation of pharmacy naloxone standing order dispensing was associated with lower community-level opioid fatality rates in Massachusetts.

## Introduction

In the US, opioid overdose caused more than 80 000 deaths in the 12 months ending in April 2023.^1^ Providing access to naloxone is a core strategy for reducing opioid fatality in the US Department of Health and Human Services’ overdose prevention strategy,^2^ the President’s national drug control strategy,^3^ and the Centers for Disease Control and Prevention’s 2022 clinical practice guidelines for prescribing opioids for pain.^4^ Legislative efforts to expand naloxone access have been pursued to enable community pharmacists to have the authority to dispense naloxone through a standing order. By July 2020, almost every state and the District of Columbia had established a mechanism allowing naloxone dispensing via standing order or similar mechanism.^5^ There is evidence that these programs significantly increase naloxone access.^6,7,8^ In March of 2023, the US Food and Drug Administration approved the first naloxone formulation for over-the-counter, nonprescription use.^9^

Despite their proliferation, there is scarce evidence on the effect of pharmacy naloxone standing orders on opioid overdose in community settings. Most evidence focused on expanded access to naloxone, such as community, pharmacy, and coprescribing overdose education and naloxone distribution (OEND) programs.^10,11,12,13^ Multiple studies on state naloxone access laws across US found reduced opioid mortality,^14,15,16^ but there was evidence that the association was driven by early adopters that passed legislation before 2011.^14^ However, a single-site interrupted time series analysis from 2012 to 2018 in Ontario, Canada, found that pharmacy-based naloxone dispensing under Ontario’s naloxone program for pharmacies was not inversely associated with overdose fatality rates.^17^ These mixed results reflect differences in study design, analytic approach, and emergence of fentanyl in the supply; yet, importantly, none focused on community-level fatality rates. Because community pharmacies can be key venues for distributing naloxone, it will be useful to not only understand the associations of these standing-order naloxone with community-level fatality rates but also infer the potential associations of over-the-counter naloxone dispensing.^9,18^

Massachusetts declared a public health emergency in response to increasing opioid overdoses in March of 2014.^19^ This declaration enabled all pharmacies in Massachusetts to voluntarily obtain a standing order to dispense naloxone kits in December 2014.^20^ The policy was strengthened on December 1, 2017, when pharmacies were mandated to obtain a standing order permitting them to distribute naloxone without prescription. In August 2018, Massachusetts General Laws, Chapter 94C, Section 19B^21^ further strengthened the policy via a single statewide standing order that authorized licensed pharmacists to dispense naloxone kits to any person at risk of experiencing an opioid-related overdose.

The standing-order naloxone policy in Massachusetts provided the opportunity to investigate its association with community-level opioid overdose. Although the rapid policy development presents challenges in identifying a single critical interruption point, pharmacy dispensing data can be used to define the inception of standing-order naloxone implementation for municipalities across Massachusetts. We hypothesized that municipalities with pharmacies dispensing standing-order naloxone would have reduced opioid fatality rates compared with municipalities that did not implement standing-order naloxone programs.

## Methods

### Study Design and Setting

This repeated cross-sectional study was reviewed and designated as not human participant research by the institutional review boards of the West Virginia University and the Boston University Medical Campus. We conducted a multisite interrupted time series analysis of quarterly opioid fatality rates comparing municipalities where community pharmacies dispensed standing-order naloxone with those did not dispense standing-order naloxone. We included data from all 351 municipalities in Massachusetts. The analysis was conducted across 24 quarters from first quarter of 2013 to fourth quarter of 2018. This study is reported following the Strengthening the Reporting of Observational Studies in Epidemiology (STROBE) reporting guideline for observational studies.

### Outcome Variable

The primary outcome was the opioid fatality rate per quarter at the municipal level. We calculated rates of opioid-related drug poisonings by community of residence using in-state deaths from the electronic database maintained by the Massachusetts Registry of Vital Records and Statistics, Massachusetts Department of Public Health. Opioid-related deaths were defined by *International Statistical Classification of Diseases and Related Health Problems, Tenth Revision *(*ICD-10*) codes indicating unintentional or undetermined intentional poisoning (code X40-X44, Y10-Y14) in the underlying cause of death field and an opioid-specific T code of T40.0-T40.4 and/or the narcotic T code T40.6 in any of the multiple cause-of-death fields.^11^

### Main Exposure Variable

We used 2 sources for retail pharmacy data of standing-order naloxone distributed between 2013 and 2018. One of the authors (A.Y.W.), was the standing-order prescriber for all major chain pharmacies, except for 1, and many independent pharmacies in Massachusetts. These standing orders required pharmacies to maintain a record of each naloxone kit dispensed and report the dates and quantities to the standing order prescriber on a monthly basis. One major chain of pharmacies used their own standing-order writer during the study period. Data from this large retail chain on standing-order naloxone dispensing have been described in prior studies.^22,23^ We merged data from both sources, which in aggregate covered 70% of retail pharmacies in Massachusetts.^24^

For the interrupted time series analysis, we used the first quarter in which standing-order naloxone was dispensed as a municipality-specific inception variable. The use of the quarter for the first standing-order naloxone dispensed captured the actual implementation inception for a given municipality. Once the pharmacies in a municipality dispensed standing-order naloxone, the municipality was coded in the exposure group and remained exposed for the duration of the study. As a result, 214 of 351 municipalities had various inceptions during the study period. The cumulative number of communities with actual standing order naloxone dispensing in each year were: 0 by 2013, 17 by 2014, 140 by 2015, 196 by 2016, 209 by 2017, and 214 by 2018. The remaining 137 municipalities did not dispense standing-order naloxone through their community pharmacies during the study period.

### Covariates

We obtained community demographics to adjust for potential municipal-level confounding. We obtained data from the American Community Survey 5-year estimates (2013-2018), including number of municipal residents, age group proportions (<25, 25-44, 45-54, 55-64, and ≥65 years), sex, race and ethnicity (African American or Black, American Indian or Alaska Native, Asian, Hispanic or Latinx, Pacific Islander, White, and other, which included multiracial and other group), high school education or less, and vacancy rate.^25^ Race and ethnicity were included because opioid use patterns differ by race and ethnicity. Vacancy rate was measured by vacant housing units per 1000 population, and vacancy rate was shown to be a marker of neighborhood distress associated with opioid overdose.^26^ To adjust for the proliferation of naloxone kits from certain other sources, we obtained the number of naloxone kits distributed to the communities by OEND programs funded by the Massachusetts Department of Public Health. We also obtained the number of naloxone kits dispensed through pharmacies that were not under a standing order (ie, with a prescription). Although these prescribed naloxone kits data were collected for the study period, the data since the second half of 2017 were incomplete. We conducted a sensitivity analysis to further adjust for prescribed naloxone kits rates. To adjust for the level of opioid treatment in the community, we obtained methadone treatment admissions rates and residential treatment admissions rates from the Massachusetts Bureau of Substance Addiction Services. In addition, the Prescription Monitoring Program provided patient rates of buprenorphine prescriptions, indicative of an opioid use disorder. These covariates were assessed in prior studies on opioid use.^11^ Given overdose risk is elevated at release from incarceration,^27^ we also obtained data on community release rates from the Massachusetts Department of Correction. Finally, because fentanyl in the illicit drug supply is the major driver of surging overdose deaths, we used the community-specific proportion of fentanyl-related deaths over all opioid deaths from Massachusetts Registry of Vital Records and Statistics as a proxy measure to control for fentanyl-related burden among these municipalities.^28^

### Statistical Analysis

We used 2-sample *t* tests to compare the municipalities that did not have standing-order naloxone vs those dispensing standing-order naloxone. For the interrupted time series, we used the quarterly municipality-specific opioid fatality counts as the units of analysis. The total units of analysis included 28 424 town-quarters (ie, 24 quarterly measures per municipality). The municipal population based on US Census estimates was used in the log scale as a population offset. We used Poisson regression models with overdispersion adjustment to model rates with a log-linear statistical model accounting for community level covariates.^11^ We used the segmented regression analysis approach^29,30^ and included multiple time series with a generalized estimation equations approach^31^ to examine the population-mean changes of level and slope of fatality rate outcome after the implementation of pharmacy standing-order naloxone dispensing. We used the equation log(Y_it_) = β_0_ + β_1_Time_it_ + β_2_Program_it_ + β_3_Time after program_it_ + BX_it_ + e_it_, where *Y_it_* represents the quarterly rate outcome at municipality *i* and time point *t*, *Time_it_* represents the time in quarter since the start of the study period, *Program_it_* is an indicator (dummy) variable representing the pharmacy standing-order naloxone dispensing program, and *Time after program_it_* represents the time in quarters after the dispensing inception date. β_0_ represents the intercept of the preprogram trend line at the index time, and B represents the coefficient vector of the municipal-level covariates set. We evaluated program outcomes based on the regression coefficients (β_2_ and β_3_) assuming the preimplementation trend remained unchanged. We examined quasilikelihood information criteria to assess goodness of fit and determine the covariance structure of the repeated quarterly measures within municipalities and the overdispersion of the Poisson distribution.^32^ We annualized β_3_ to represent the change of rate per year after implementation of pharmacy standing-order naloxone programs.

To ascertain robustness of our findings, we conducted 5 sensitivity analyses. First, instead of using the concurrent quarter as the first dispensing quarter, we applied 1-quarter lag by using the subsequent quarter as the starting quarter, because the date of the first standing-order naloxone dispensed may not fall at the beginning of a quarter. Second, we removed substance use disorder treatment variables (eg, methadone admission rate, residential admission rate, and patient rate of buprenorphine prescription for opioid use disorder) that could be potential mediators. Third, we further controlled for prescribed pharmacy-based naloxone kit distribution rates that could partially affect opioid overdose rates in these municipalities. Fourth, to account for potential unmeasured health system effects, we followed a prior method^10^ and refit the adjusted Poisson fatal overdose models replacing fatal death rates with opioid death to cancer death rate ratios for each municipality-quarter unit using data from the Massachusetts Registry of Vital Records and Statistics. We used *ICD-10* codes representing malignant neoplasms (codes C000-C979) to define cancer deaths. Cancer death rate served as a potential control of the underlying health system change over the study period; thus, a significant association with the opioid-to-cancer–death rate ratio outcome would indicate that opioid deaths were associated with the standing-order naloxone program after accounting for the underlying health system changes. Fifth, because the Massachusetts state mandate for standing-order naloxone dispensing took effect in August 2018, we restricted the final sensitivity analysis to a shortened study period from the first quarter of 2013 to the second quarter of 2018 (ie, 22 quarters).

All tests used a significance level of P ≤ .05, and all *P* values were 2-sided. All analyses were conducted using SAS software version 9.4 (SAS Institute). Data were analyzed from December 2021 to November 2023.

## Results

The mean (SD) population size across 351 municipalities was 18 818 (39 368; median [IQR], 10 314 [3635 to 21 781]), the mean (SD) proportion of female individuals was 51.1% (2.8 percentage points), and the mean (SD) age proportions were 29.2% (5.7 percentage points) younger than 25 years, 22.7% (4.8 percentage points) aged 25 to 44 years, 17.2% (3.0 percentage points) aged 45 to 54 years, 15.3% (3.9 percentage points) aged 55 to 64 years, and 15.7% (5.1 percentage points) aged 65 years or older. Pharmacies from 214 municipalities (60.9%) reported ever dispensing standing-order naloxone over the study period. The implementation of standing-order naloxone programs was mapped with the opioid fatality rates (eFigure in Supplement 1).

At the baseline of the first quarter of 2013, municipalities that eventually had standing-order naloxone dispensed in their pharmacies had greater proportions of younger, female, African American, Asian, Hispanic, or other race residents and greater rates of residential admissions compared with municipalities without standing-order naloxone dispensed in their pharmacies (Table 1). Municipalities that eventually had standing-order naloxone dispensed had larger population size and lower vacancy rate (Table 1) and a higher quarterly opioid fatality rate at baseline compared with municipalities that did not have standing-order naloxone dispensing (3.51 vs 1.03 per 100 000 population; *P* < .001) (Table 1).

**Table 1.** Comparison of Characteristics of 351 Municipalities in Massachusetts in the First Quarter of 2013 and the Fourth Quarter of 2018

Characteristic	First quarter of 2013			Fourth quarter of 2018			
	Municipalities, mean (SD)		*t *test (*P* value)	Municipalities, mean (SD)		*t* test (*P* value)
	No NSO (n = 137)	Eventual NSO (n = 214)^a^		No NSO (n = 137)	With NSO (n = 214)	
Quarterly opioid fatality rate, No. per 100 000 population	1.03 (4.46)	3.51 (6.30)	−4.01 (<.001)	4.17 (10.56)	5.66 (6.90)	−1.61 (.11)
Age, y, %						
<25	27.49 (6.30)	30.23 (4.99)	−4.52 (<.001)	26.15 (6.39)	29.08 (5.38)	−4.61 (<.001)
25-44	20.61 (4.31)	23.99 (4.70)	−6.79 (<.001)	19.53 (4.45)	23.44 (4.96)	−7.50 (<.001)
45-54	18.15 (3.17)	16.52 (2.72)	5.13 (<.001)	15.68 (3.46)	15.04 (2.38)	2.06 (.04)
55-64	17.36 (4.42)	13.91 (2.70)	9.07 (<.001)	18.12 (4.08)	14.71 (2.75)	9.32 (<.001)
≥65	16.39 (5.88)	15.35 (4.54)	1.87 (.06)	20.52 (6.45)	17.73 (5.38)	4.38 (<.001)
Sex, %						
Female	50.59 (3.16)	51.35 (2.45)	−2.53 (.01)	50.39 (3.50)	51.35 (2.36)	−3.07 (.002)
Male	49.41 (3.16)	48.65 (2.45)	2.53 (.01)	49.61 (3.50)	48.65 (2.37)	3.07 (.002)
Race and ethnicity, %						
African American or Black	1.02 (1.68)	3.06 (5.04)	−4.57 (<.001)	1.41 (2.13)	3.54 (5.17)	−4.59 (<.001)
American Indian or Alaska Native	0.51 (3.58)	0.14 (0.22)	1.49 (0.14)	0.31 (1.79)	0.16 (0.37)	1.22 (.22)
Asian	1.46 (2.16)	3.90 (4.63)	−5.79 (<.001)	1.74 (2.99)	4.57 (5.51)	−5.51 (<.001)
Hispanic or Latinx	1.92 (1.93)	5.64 (8.87)	−4.85 (<.001)	2.60 (2.07)	6.67 (9.88)	−4.75 (<.001)
Pacific Islander	0.01 (0.06)	0.02 (0.07)	−1.57 (.12)	0.05 (0.19)	0.03 (0.07)	1.60 (.11)
White	94.87 (5.92)	88.73 (10.63)	6.17 (<.001)	93.92 (5.70)	87.03 (10.93)	6.81 (<.001)
Other^b^	2.14 (2.14)	4.15 (5.49)	−4.09 (<.001)	2.56 (2.15)	4.68 (2.15)	−4.58 (<.001)
Educational level, %						
≤High school	33.01 (11.38)	34.11 (12.77)	−0.82 (.41)	31.14 (11.22)	31.58 (12.41)	−0.34 (.74)
≥College	69.99 (11.38)	65.89 (12.77)	0.82 (.41)	68.86 (11.22)	68.42 (12.41)	0.34 (.74)
Vacancy units, No. per 1000 population	142.3 (262.9)	58.22 (128.3)	4.00 (<.001)	173.4 (447.8)	61.08 (137.1)	3.43 (<.001)
Naloxone kits distributed by OEND program, No. per 1000 population	0.10 (0.20)	0.20 (0.21)	−4.51 (<.001)	0.42 (0.64)	0.83 (1.20)	−3.66 (<.001)
Releases from incarceration, No. per 1000 population	0.03 (0.10)	0.09 (0.11)	−5.06 (<.001)	0.04 (0.11)	0.07 (0.09)	−3.49 (<.001)
BSAS methadone admissions, No. per 1000 population	0.26 (0.43)	0.30 (0.44)	−0.88 (.38)	0.18 (0.38)	0.27 (0.36)	−2.36 (.02)
BSAS residential admissions, No. per 1000 population	0.94 (0.93)	1.57 (1.07)	−5.63 (<.001)	0.76 (0.78)	1.57 (1.32)	−6.42 (<.001)
Buprenorphine prescription for OUD, No. per 1000 population	6.06 (3.86)	7.10 (6.02)	−1.80 (.07)	8.28 (5.95)	9.56 (8.23)	−1.58 (.12)
Fentanyl-related deaths among opioid-related deaths, %	2.19 (14.69)	5.01 (20.01)	−1.42 (.16)	16.06 (36.35)	52.06 (48.44)	−7.46 (<.001)
Cancer-related death rate, No. per 100 000 population	35.65 (47.80)	40.88 (21.22)	−1.40 (.16)	35.98 (44.82)	41.19 (23.90)	−1.41 (.16)
Municipal population, No.						
Mean	4117 (3342)	28 230 (48 136)	−5.85 (<.001)	4239 (3515)	29 204 (51 373)	−5.68 (<.001)
Median	3245 (1376-6211)	17 646 (10 857-29 968)	NA	3292 (1380-6551)	18 099 (11 301-31 177)	NA

Abbreviations: BSAS, Massachusetts Bureau of Substance Addiction Services; NA, not applicable; NSO, naloxone standing order; OEND, opioid education and naloxone distribution; OUD, opioid use disorder.

^a^ Massachusetts has 351 municipalities. During the period from 2013 to 2018, pharmacies from 214 municipalities used NSO to dispense naloxone kits, while 137 municipalities did not. The 214 municipalities had various inception of actual standing-order naloxone dispensing during the study period. The cumulative number of communities with actual standing-order naloxone dispensing in each year were: 1 by 2013, 17 by 2014, 140 by 2015, 196 by 2016, 209 by 2017, and 214 by 2018.

^b^ Other race category included multiracial and other race groups.

Table 2 summarizes the changes in municipal opioid fatality rates after the implementation of standing-order naloxone dispensing from 2013 to 2018. Adjusting for municipality-level covariates and the trend of opioid fatality prior to standing-order naloxone dispensing, we did not detect a statistically significant level change in the opioid fatality rate (adjusted rate ratio [aRR], 1.09; 95% CI, 0.99 to 1.20; *P* = .08) after naloxone dispensing under the standing-order naloxone commenced; however, there was a significant slope decrease (annualized aRR, 0.84; 95% CI, 0.78 to 0.91; *P* < .001). The decrease of slope corresponded to an annual relative decrease of 16.0% of the opioid fatality rate per 100 000 population. No differential trends were detected (β = −0.010; 95% CI, −0.028 to 0.008; *P* = .26), thus meeting the assumption of parallel baseline trends in municipalities with and without standing-order naloxone.^33^ Municipalities with greater proportions of residents with only high school education or less, greater rates of inpatient residential admissions, more patients with buprenorphine prescriptions per population, and a greater proportion of fentanyl-involved opioid deaths had increased opioid fatality rates (Table 3).

**Table 2.** Changes in Level and Slope of Opioid Fatality Rates After Implementation^a^ of Naloxone Standing Order Across 351 Municipalities in Massachusetts From the First Quarter of 2013 to the Fourth Quarter of 2018

Opioid fatality rate	Unadjusted model		Main adjusted model	
	Rate ratio (95% CI)	*P* value	Adjusted rate ratio (95% CI)^b^	*P* value
Level change	1.31 (1.22-1.41)	<.001	1.09 (0.99-1.20)	.08
Slope change (annualized per year)	0.91 (0.85-0.96)	.002	0.84 (0.78-0.91)	<.001

^a^ The exposure of the policy implementation was based on the actual starting quarter when the municipality had their first standing-order naloxone dispensed. Therefore, the actual starting quarter varied across the 214 municipalities that had standing-order naloxone dispensed in their pharmacies. Once the pharmacies in a municipality dispensed standing-order naloxone, the municipality was coded in the exposure condition. This analysis also included the 137 municipalities that did not dispense standing-order naloxone as comparative series.

^b^ The adjusted model accounted for municipal-level factors of age groups, male sex, race and ethnicity, education level, vacancy units, naloxone kits distributed by Massachusetts’ opioid education and naloxone distribution program, releases from incarceration, Massachusetts Bureau of Substance Addiction Services methadone treatment admissions and residential admissions, Massachusetts Prescription Monitoring Program patient rate of buprenorphine prescriptions indicated for opioid use disorder, and proportion of fentanyl-related death.

**Table 3.** Interrupted Time Series Poisson Regression Model of the Associations of Implementing Standing Order Naloxone Dispensing With Opioid Fatality Rate Across 351 Municipalities in Massachusetts From the First Quarter of 2013 to the Fourth Quarter of 2018

Opioid fatality rate^b^	Main adjusted model^a^	
	β (95% CI)^c^	*P* value
Exposure variables		
Intercept	−11.45 (−13.28 to −9.621)	<.001
Change in the outcome level after implementation	0.085 (−0.011 to 0.182)	.08
Change in trend per quarter after implementation	−0.044 (−0.064 to −0.024)	<.001
Trend per quarter before implementation	0.012 (0.003 to 0.021)	.01
Age, y, %		
25-44	0.001 (−0.014 to 0.014)	.96
45-54	−0.003 (−0.034 to 0.029)	.09
55-64	0.013 (−0.009 to 0.034)	.25
≥65	0.001 (−0.012 to 0.012)	.99
Female sex, %	−0.006 (−0.025 to 0.013)	.53
Race and ethnicity, %		
African American or Black	−0.003 (−0.016 to 0.010)	.66
American Indian or Alaska Native	0.022 (−0.023 to 0.066)	.34
Asian	0.001 (−0.014 to 0.016)	.92
Hispanic or Latinx	−0.006 (−0.013 to 0.002)	.16
Pacific Islander	−0.003 (−0.352 to 0.346)	.99
White	−0.003 (−0.014 to 0.009)	.67
≤High school education, %	0.018 (0.013 to 0.023)	<.001
Vacancy units, per 1000 population	0.0004 (−0.001 to 0.001)	.12
Naloxone kits distributed by OEND program, per 1000 population	−0.036 (−0.109 to 0.038)	.34
Releases from incarceration, per 1000 population	−0.008 (−0.226 to 0.209)	.94
BSAS methadone admissions, per 1000 population	−0.040 (−0.158 to 0.079)	.51
BSAS residential admissions, per 1000 population	0.104 (0.055 to 0.154)	<.001
Patients with buprenorphine prescription for OUD, per 1000 population	0.015 (0.005 to 0.025)	.003
Fentanyl-related deaths among opioid-related deaths, %	0.011 (0.009 to 0.013)	<.001

Abbreviations: BSAS, Massachusetts Bureau of Substance Addiction Services; OEND, opioid education and naloxone distribution; OUD, opioid use disorder.

^a^ The exposure of the policy implementation was based on the actual starting quarter when the municipality had their first standing order naloxone dispensed. Therefore, the actual starting quarter varied across the 214 municipalities that had standing order naloxone dispensed in their pharmacies. Once the pharmacies in a municipality dispensed standing order naloxone, the municipality was coded in the exposure condition. This analysis also included the 137 municipalities without standing-order naloxone as comparative series.

^b^ Municipality quarter data of opioid fatality rate per 100 000 population were available from the first quarter of 2013 to the fourth quarter of 2018.

^c^ The adjusted model accounted for municipal-level factors of age groups, male sex, race and ethnicity, education, vacancy units, naloxone kits distributed by Massachusetts’ OEND program, releases from incarceration, BSAS methadone treatment admissions and residential admissions, Massachusetts Prescription Monitoring Program patient rate of buprenorphine prescriptions indicated for opioid use disorder, and proportion of fentanyl-related death among opioid-related deaths.

All sensitivity analyses yielded consistent results with respect to no statistically significant level changes yet significant slope changes of opioid fatality rates (Table 4). First, lag-1 analysis also detected slope change (annualized aRR, 0.84; 95% CI, 0.77 to 0.91; *P* < .001). Statistically significant slope changes were also detected in models when removing municipal substance use disorder treatment utilization (aRR, 0.85; 95% CI, 0.78 to 0.92; *P* < .001) and additionally controlling for prescribed pharmacy-based naloxone kit rates (aRR, 0.84; 95% CI, 0.78 to 0.91; *P* < .001). In the fourth sensitivity analysis, the opioid-related overdose fatality outcomes were substituted for the ratio of opioid death rates over the cancer-related death rates. Slope change was also detected (aRR, 0.85; 95% CI, 0.81 to 0.90; *P* < .001). Thus, the decrease of slope in opioid fatality rates after standing-order naloxone programs was independent of underlying health system change over the study period. Finally, excluding the last 2 quarters of 2018 still resulted in a significant slope change for the opioid fatality rate (aRR, 0.87; 95% CI, 0.80 to 0.94; *P* < .001).

**Table 4.** Post Hoc Sensitivity Analyses of the Associations of Implementing Standing-Order Naloxone Dispensing With Opioid Fatality Rate Across 351 Municipalities in Massachusetts From the First Quarter of 2013 to the Fourth Quarter of 2018

Opioid fatality rate^b^	Main adjusted model^a^	
	Adjusted rate ratio (95% CI)^c^	*P* value
**Lag-1 approach using the following quarter as the starting quarter**		
Level change	1.08 (0.98-1.18)	.13
Slope change (annualized per year)	0.84 (0.77-0.91)	<.001
**Remove potential mediators (eg, substance use disorder treatment variables)**		
Level change	1.08 (0.99-1.17)	.07
Slope change (annualized per year)	0.85 (0.78-0.92)	<.001
**Include pharmacy-based non–standing order prescribed naloxone kits rate as additional covariate**		
Level change	1.09 (0.99-1.20)	.08
Slope change (annualized per year)	0.84 (0.78-0.91)	<.001
**Based on the rate ratio of opioid death rate over cancer death rate**		
Level change	0.96 (0.85-1.10)	.57
Slope change (annualized per year)	0.85 (0.81-0.90)	<.001
**Restrict study period from first quarter 2013 to second quarter 2018**		
Level change	1.07 (0.97-1.17)	.15
Slope change (annualized per year)	0.87 (0.80-0.94)	<.001

^a^ The exposure of the policy implementation was based on the first standing order naloxone dispensed quarter. Therefore, the actual starting quarter varied across the 214 municipalities that had standing order naloxone dispensed in their pharmacies. This analysis also included the 137 municipalities without standing-order naloxone dispensing as comparative series.

^b^ Municipality quarter data of opioid fatality rate per 100 000 population were available from the first quarter of 2013 to the fourth quarter of 2018.

^c^ The adjusted model accounted for municipal-level factors of age groups, male sex, race and ethnicity, education, vacancy units, naloxone kits distributed by Massachusetts’ opioid education and naloxone distribution program, releases from incarceration, Massachusetts Bureau of Substance Addiction Services methadone treatment admissions and residential admissions, Massachusetts Prescription Monitoring Program patient rate of buprenorphine prescriptions indicated for opioid use disorder, and proportion of fentanyl-related death among opioid-related deaths.

## Discussion

This repeated cross-sectional study with interrupted time series analysis found that despite an uptrend of opioid fatality rate across Massachusetts, standing-order naloxone dispensing was associated with a relative, gradual, and significant decrease in opioid fatality rates in the municipalities with standing-order naloxone dispensing compared with the municipalities where pharmacies did not dispense standing-order naloxone. Our main analysis and several sensitivity analyses confirmed the association with reduced community-level opioid fatality rates.

Although we observed a reduction in the slope of opioid fatality rates over time, we did not detect an immediate change of opioid fatality rates in the adjusted models. This was true whether we anchored the policy implementation on the first standing-order naloxone dispensing event or lagged it by 1 quarter. It is possible that community pharmacies started dispensing naloxone in response to heightened opioid overdose in the communities. The lack of immediate level change was also expected, given the gradual process to make community residents aware of the standing-order prescribing of naloxone in their community pharmacies. Detecting an immediate policy impact can be challenging when policy implementation is shaped by contextual factors and time-delayed effects in a fluid and complex ecological system.^34^ Policy-, chain- and pharmacy-level interventions that occurred during the period of study are worth considering. For example, a multicomponent study examined barriers experienced by patients, caregivers, and pharmacists^35,36,37,38^; developed educational intervention materials tailored to these barriers for the pharmacy and staff^39,40^; and then implemented the materials through academic detailing visits.^41^ Such efforts may have boosted the standing-order naloxone outcomes through systemic dissemination and implementation in community pharmacies.

Municipalities with greater proportions of residents with lower education had higher opioid fatality rates. A 2020 study found that individuals with only high school education or less were more likely to die of opioid overdose compared with individuals with a graduate degree.^42^ This association of overdose fatality with lower education likely reflects the health impacts from structural inequities in social resources, like education.

This study conducted a multisite interrupted time series using standing-order naloxone dispensing as a natural experiment. The use of a community’s actual quarter of their first standing order naloxone dispensing also departs from prior approaches that relied on between-state naloxone access law pre-post comparison,^14,15,16^ thus yielding new evidence for the association of pharmacy naloxone dispensing with opioid fatality at the community level.

### Limitations

The study has several limitations. First, our study is susceptible to residual confounding, particularly other unmeasured community-level factors. We tried to mitigate confounding by including a large set of community-level covariates. Second, although our data covered 70% of retail pharmacies in Massachusetts and should have correctly identified all pharmacies dispensing standing-order naloxone prior to state mandate in October 2018, it is possible that some pharmacies dispensed standing-order naloxone under standing orders not covered by our data. If this happened, it was likely a small proportion. A 2023 study comparing a national commercial pharmacy naloxone dataset with the same pharmacy naloxone data sources in Massachusetts used in this study found that the overall (standing order and non–standing order combined) dispensing counts were similar with the commercial dataset, though the commercial dataset counted more overall naloxone dispensed in the later quarters.^43^ However, the commercial dataset had no indicator for standing-order naloxone, so it is not possible to test our hypothesis with the commercial dataset. Third, we were not able to measure or include in our model coprescribing of naloxone with prescription opioids, which has been shown in multiple states to drive pharmacy naloxone dispensing,^44,45^ because it was not a policy mandate in Massachusetts during this time. Fourth, we only used the first standing-order naloxone dispensing quarter as the start of the intervention, and we did not capture the actual volume of standing-order naloxone per quarter during the study period. Fifth, this study also did not directly measure over-the-counter naloxone, as it was not available. However, standing-order naloxone dispensing is a close approximation of over-the-counter naloxone. We did not investigate price of naloxone as a factor in this study, as we did not have price information. A prior study showed that insurance coverage for naloxone during this period was common.^20^

## Conclusions

The findings of this study with interrupted time series analysis provide support for implementation of standing-order naloxone dispensing and expansion of access to naloxone to address opioid overdose crisis. Our findings are also consistent with previous literature refuting the idea of naloxone as a moral hazard—that naloxone provision would lead to increased opioid use or overdose.^13,46,47,48^ With the US Food and Drug Administration’s approval of certain over-the-counter naloxone products,^9^ it is important to examine their diffusion across communities over time, augmentation of other formulations through existing standing-order mandates, and ultimately their impact on reducing opioid fatalities. Future research could ascertain how communities of color (eg, African American or Black, American Indian or Alaska Native, Asian, Hispanic or Latinx, Pacific Islander) or other vulnerability characteristics, such as low economic resources, could benefit from proactive, broader access to naloxone that comes from policies like standing orders or over-the-counter to overcome other barriers, including willingness to engage with first responders and readiness to administer naloxone. It would also be valuable to examine how standing-order naloxone dispensing may interact with other strategies, such as medications for opioid use disorder and referrals to recovery support services, in reducing opioid overdose.

## Notes

Supplement 1. **eFigure.** Change of Opioid Fatality Rate and Uptake of Pharmacy Naloxone Standing Order Implementation Across 351 Municipalities in Massachusetts From the First Quarter of 2013 to the Fourth Quarter of 2018

Supplement 2.
Data Sharing Statement
