# Supplementary material for: Pharmacy Naloxone Standing Order and Community Opioid Fatality Rates Over Time
Source: JAMA Netw Open. 2024 Aug 29;7(8):e2427236. doi: 10.1001/jamanetworkopen.2024.27236 (PMC11362859; doi:10.1001/jamanetworkopen.2024.27236)
Supplement: Supplement 1. — eFigure. Change of Opioid Fatality Rate and Uptake of Pharmacy Naloxone Standing Order Implementation Across 351 Municipalities in Massachusetts From the First Quarter of 2013 to the Fourth Quarter of 2018 [file jamanetwopen-e2427236-s001.pdf]

Supplemental Online Content

Xuan Z, Walley AY, Yan S, Chatterjee A, Green TG, Pollini RA. Pharmacy naloxone standing order and community opioid fatality rates over time. *JAMA Netw Open*. 2024;7(8):e2427236. doi:10.1001/jamanetworkopen.2024.27236

eFigure. Change of Opioid Fatality Rate and Uptake of Pharmacy Naloxone Standing Order Implementation Across 351 Municipalities in Massachusetts From the First Quarter of 2013 to the Fourth Quarter of 2018

This supplemental material has been provided by the authors to give readers additional information about their work.

© 2024 Xuan Z et al. *JAMA Network Open.*

eFigure. Change of Opioid Fatality Rate and Uptake of Pharmacy Naloxone Standing Order Implementation Across 351 Municipalities in Massachusetts from the First Quarter of 2013 to the Fourth Quarter of 2018

Note: 2 (column) by 6 (row) figure with the left column showing the cumulative status (as measured by the number of quarters since the 1st pharmacy NSO dispensed) and the right column showing the model-based predicted opioid fatality rate at municipality level.

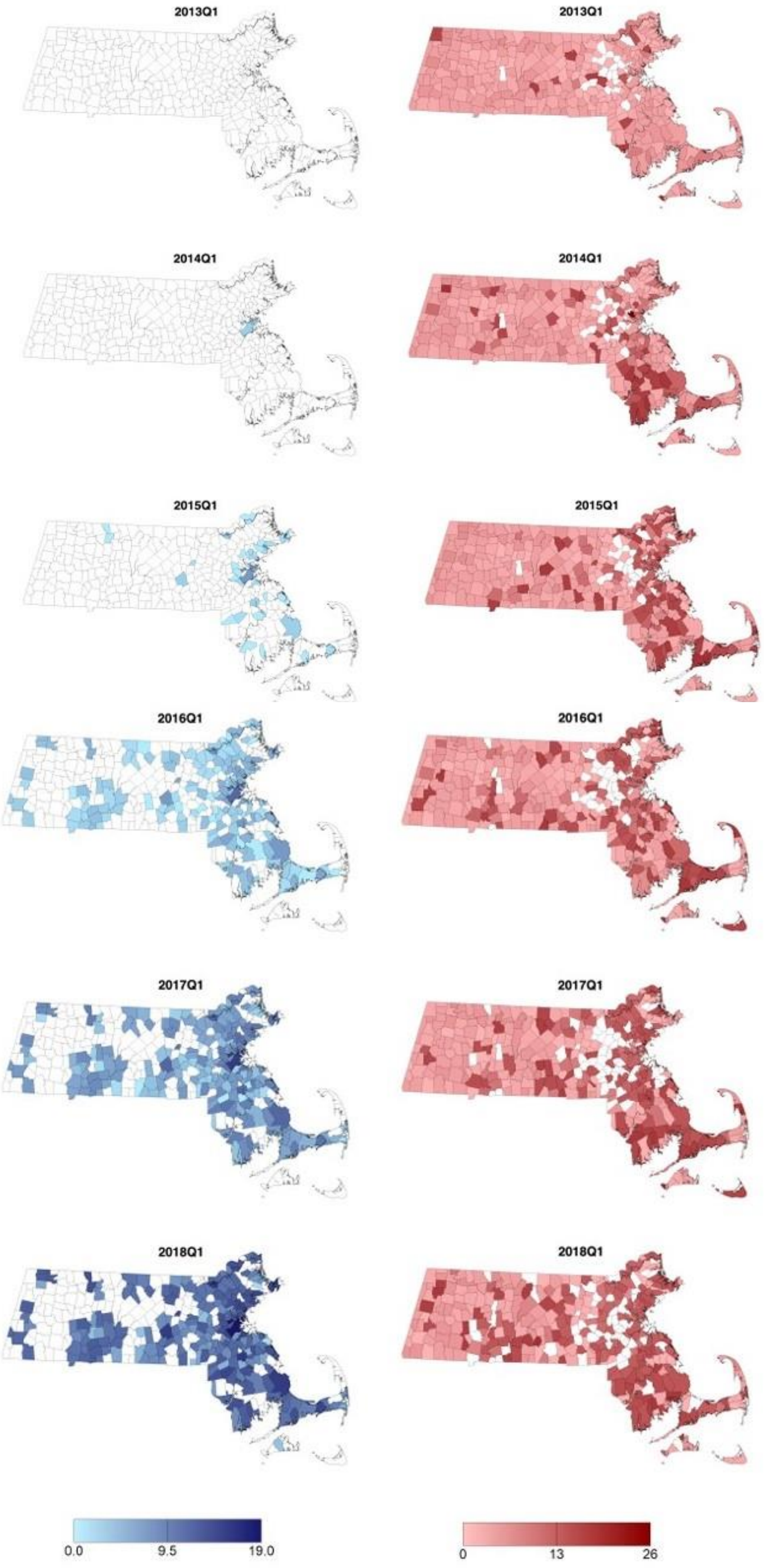

© 2024 Xuan Z et al. *JAMA Network Open.*
