# Supplementary material for: Pharmacy Naloxone Standing Order and Community Opioid Fatality Rates Over Time
Source: JAMA Netw Open. 2024 Aug 29;7(8):e2427236. doi: 10.1001/jamanetworkopen.2024.27236 (PMC11362859; doi:10.1001/jamanetworkopen.2024.27236)
Supplement: Supplement 2. — Data Sharing Statement [file jamanetwopen-e2427236-s002.pdf]

Data Sharing Statement

Xuan. Pharmacy Naloxone Standing Order and Community Opioid Fatality Rates Over Time. *JAMA Netw Open*. Published August 29, 2024. doi:10.1001/jamanetworkopen.2024.27236

Data
Data available: Yes
Data types: Other (please specify)
Additional Information: Pharmacy prescription data
How to access data: Per data use agreement from investigators
When available: With publication

Supporting Documents
Document types: Statistical/analytic code
How to access documents: Per user agreement
When available: With publication

Additional Information
Who can access the data: Researchers whose proposed use of the data has been approved
Types of analyses: For research purpose
Mechanisms of data availability: with a signed data access agreement
